# Integrated control of braking-yaw-roll stability under steering-braking conditions

**DOI:** 10.1038/s41598-023-48535-1

**Published:** 2023-11-30

**Authors:** Jia Chen, Yihang Liu, Renping Liu, Feng Xiao, Jian Huang

**Affiliations:** 1 Automotive Engineering School, Chengdu Aeronautic Polytechnic, Chengdu, 610100 China; 2 College of Mechanical and Vehicle Engineering, Chongqing University, Chongqing, 400044 China

**Keywords:** Mechanical engineering, Applied mathematics

## Abstract

Sharp steering-braking at a high speed exposes sport utility vehicles with high gravity centers and narrow wheel tracks to the risks of tire locking, sideslip and rollover. To avoid these risks and ensure braking safety, yaw stability and roll stability upon steering-braking, a braking-yaw-roll stability integrated control strategy was proposed, which consists of a supervisor, an upper and a lower controller for the front and rear axle independent drive electric vehicle. In the supervisor, a nonlinear vehicle predictive model was constructed and four control modes were proposed according to the vehicle status and rollover indexes. The weight coefficients between braking force, yaw stability and roll stability are determined dynamically by the control mode and output to the upper controller. The upper controller used a nonlinear model predictive control to determine the longitudinal braking force distribution of the four wheels. And in the lower controller, the regenerative braking torque and friction braking torque of each wheel were distributed. Finally, simulation verifications were carried out on the high and low adhesion roads. The results show that the control strategy proposed in this study can effectively prevent the vehicle from rollover while ensuring braking safety and yaw stability.

## Introduction

The development of automobile industry has a history of hundreds of years. The design of automobiles has evolved from simplicity to complexity, from basic to profound. Their various performance aspects have continuously improved, making them an indispensable means of transportation in many people’s lives. With the development of automotive electronic technology and people’s increasing emphasis on traffic safety, various vehicle active safety systems have gradually emerged. They can adapt vehicles to various driving conditions and road environments to improve the active safety performance. Among the vehicle active safety systems, the most typical and first widely used is the wheel anti-lock braking system (ABS)^1^, which has a presence in abundant literature. Under the condition of critical braking or low road adhesion, ABS can prevent the wheel from locking by constantly adjusting the braking torque, so that the maximum longitudinal braking force of the wheels can be obtained. Zhang et al.^2^ obtained the optimal slip ratio of wheels by estimating the road adhesion coefficient and designed a sliding mode controller to make the wheels follow the optimal slip ratio. Xu et al.^3^ calculated the real-time ratio of wheel longitudinal force change rate to slip ratio change rate, so that the maximum tire longitudinal force can be obtained with unknown road adhesion coefficient. For the vehicle dynamic model, Min et al.^4^ used a particular vehicle inverse dynamics model to calculate the required torque and steering control for trajectory tracking, which leads to a better safety and energy consumption performance. Li et al.^5^ optimized the distribution of regenerative braking torque and friction braking torque, improved the following accuracy of the actual braking torque relative to the target braking torque and reduced the change frequency of the regenerative/friction braking torque.

The conventional ABS only controls the longitudinal force of wheels to realize the braking safety in straight driving condition. To achieve active safety performance of vehicles under some complicated conditions such as steering, electronic stability programs (ESPs) have begun to gain popularity in automobiles^6^. Current ESPs can be divided roughly into two categories, i.e. direct yaw moment control (DYC) and active front and rear wheel steering system (AFS/ARS). DYC is based on the differential braking and driving concept, and compensates the vehicle's required yaw moment with the extra yaw moment formed by different braking/driving forces of each wheel on both sides to make the driving path follow the driver’s intention. AFS/ARS realizes the control of the yaw moment by providing an additional angle to the front/rear wheels. In literatures^7–10^, a yaw stability control strategy with layered structure is adopted, in which the upper structure calculates the required yaw moment and the lower structure distributes the driving/braking torque to the four wheels based on the consideration factors including tire workload, additional yaw moment, tire longitudinal force following deviation, etc. To solve the chattering problem of traditional sliding mode control, Xie et al.^11^ designed an active rear-wheel steering system and direct yaw moment cooperative control system to improve vehicle handling stability, and used a fuzzy controller optimized by the genetic algorithm to output compensated yaw moment for vehicle stability. Wang et al.^12^ designed a cooperative control strategy of differential drive assisted steering and direct yaw moment control, which could improve the handling stability of a vehicle in a variety of typical conditions according to simulation.

The ABS and ESP systems are solutions to secure the longitudinal and lateral active safety of vehicles, but for vehicles with high gravity centers and narrow wheel-tracks, such as sport utility vehicles (SUVs), rollover is also a major safety hazard. In terms of rollover warning, Larish et al.^13^ proposed a predictive lateral load transfer ratio (PLTR) algorithm and experimentally verified that the PLTR outperforms the traditional load transfer ratio (LTR). In terms of rollover prevention control, there is a wide spectrum of studies on driving/braking torque distribution system^14,15^, active steering system^16–18^ and active suspension system^19–21^, which may serve as effective solutions to vehicle rollover. As for the load transfer, Luo et al.^22^ proposed a new preconditioned modified conjugate gradient algorithm based on improved gradient operator and preconditioned technology for moving force identification, which is proved to be a stable and reliable identification method for static and low-frequency components.

All the above studies are related to independent control of braking, yaw motion or roll motion. It is found that all may take braking/driving torque as the control input. Then there is a strong coupling effect between the control of braking, yaw motion and roll motion. Considering the independent and controllable braking torque on the four wheels, many scholars have done a lot of research on the integrated control of braking, yaw and roll, with the intent to improve the vehicle braking safety, yaw stability and roll stability. Zhu et al.^23^ put forward a rollover warning algorithm based on a neural network and used model predictive control (MPC) for coordinated control of the AFS-DYC integrated rollover prevention system, which improved the accuracy of vehicle rollover warning and lateral stability. Environmental perception is also a prerequisite for vehicle driving safety. Based on the optimization of lidar and camera, Han et al.^24^ proposed several constraint conditions based on the fusion of the two data and predicted the location of missing lane lines by using the road information identified by lidar and image, which leads to a better performance than existing method. As for multi-objective optimization problems, Cao et al.^25^ constructed a many-objective optimization model of multi-depot heterogeneous-vehicle and tackle the model through a memetic algorithm based on Two_Arch2, which effectively optimized the many-objective model. Lee et al.^26^ proposed a switching MPC controller to track the desired path while preventing rollover through differential braking and active rear wheel steering. Jo et al.^27^ proposed a vehicle chassis control system that arranges the control priorities in the following order according to the degree of danger of various instability conditions: roll stability control, yaw stability control, excessive/understeer control. Zhao et al.^28^ considered both the sprung and unsprung masses of the vehicle and used the H∞ controller to integrate the AFS system and DYC system. The simulation results show that the integrated controller can simultaneously ensure the yaw and roll stability of the vehicle. Li et al.^29^ established a nonlinear three-degree-of-freedom vehicle stability controller with MPC and experimentally proved that the controller works well to secure vehicle yaw and roll stability under complex steering conditions. In literature^30–32^, integrated control of ABS and yaw stability under critical steering-braking condition was realized by reducing the braking torque on wheels of one side to compensate the desired yaw moment based on the differential braking principle.

In summary, current research on vehicle active safety systems can be divided into independent control and integrated control. Independent control mainly focuses on one of braking safety, yaw stability and roll stability. The integrated control mainly focuses on the integration of brake-yaw or roll-yaw. However, upon sharp steering-braking at a high speed, SUVs with high gravity centers and narrow wheel tracks are still exposed to the risks of tire locking, sideslip and rollover. To alleviate this problem, it is necessary to simultaneously consider braking safety, yaw stability and roll stability. The main contributions of this study can be concluded as follows: (1) A braking-yaw-roll integrated control (BYRIC) strategy was proposed to ensure vehicle braking safety, yaw stability and roll stability. (2) A distribution strategy for regenerative braking torque and friction braking torque was proposed. In the case of non-emergency braking, regenerative braking works and reduces the vehicle energy consumption. (3) Under the condition of high/low road adhesion coefficient, compared with the conventional ABS control (CAC), the proposed control strategy could effectively prevent the vehicle from rollover while ensuring braking safety and yaw stability.

The rest of this study is organized as follows: In Sect. “Vehicle model”, a vehicle body dynamics model including longitudinal, lateral, yaw and roll motion was established. The BYRIC strategy was proposed in Sect. “Braking-yaw-roll integrated control strategy”. In Sect. “Simulation results”, simulation verification of BYRIC is carried out. Finally, the conclusion is drawn.

## Vehicle model

**Figure 1:**
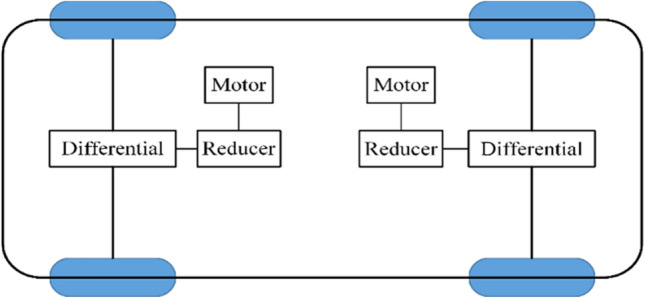
Structure of the FRID-EV.

The front and rear axle independent drive electric vehicle (FRID-EV) in this study is structured as shown in Fig. 1. The front and rear axles are driven by two motors independently, the power of the motors is transmitted to the front and rear wheels through a reducer and differential respectively.

### Vehicle body model

**Figure 2:**
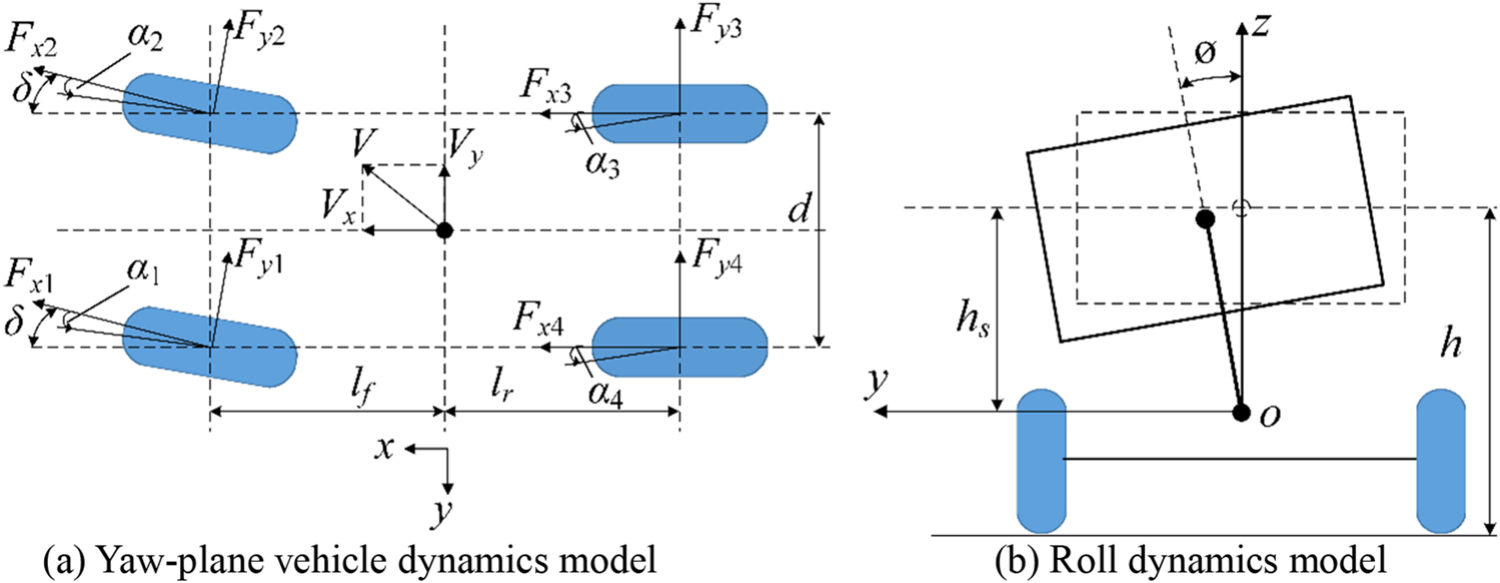
Vehicle body model.

To study the influence of braking, yaw and roll motion on vehicle stability, a vehicle body dynamics model including longitudinal, lateral, yaw and roll motion was established as shown in Fig. 2. Among them, longitudinal and lateral motions are the most visually apparent aspects of a vehicle’s movement. While steering, the vehicle also undergoes a yaw movement about the vertical axis and a roll movement about the longitudinal axis. These two movements greatly affect the safety and comfort of a vehicle. In particular, once the movement in these two dimensions exceeds the limit, vehicle sideslip and rollover might occur, which is very dangerous. A typical front and rear axle independent drive electric SUV referenced from the software CarSim was taken as the research object. The main parameters of the vehicle were shown in Table 1.

**Table 1:** Simulation parameters.

Parameters	Value	Unit
Vehicle mass ($m$)	1171	kg
Wheel-track ($d$)	1481	mm
Distance between front axle and center of gravity (${l}_{f}$)	1040	mm
Distance between rear axle and center of gravity (${l}_{r}$)	1560	mm
Motor peak torque (${T}_{max}$)	90	N
Motor peak power (${P}_{max}$)	50	kw
Motor rated speed (${n}_{e}$)	5500	r/min
Rollover threshold (${LTR}_{th}$)	0.8	–
Reaching law parameter ($\varepsilon$)	0.02	–
Reaching law parameter (${k}_{d}$)	50	–

According to Newton’s second law and the principle of moment balance, the dynamic equations for the vehicle in the longitudinal, lateral, yaw and roll dimensions are expressed as follows:

1 $$m\left({\dot{V}}_{x}-r{V}_{y}\right)+{m}_{s}{h}_{s}\ddot{\mathrm{\varnothing }}=\left({F}_{x1}+{F}_{x2}\right)\mathrm{cos}\delta -\left({F}_{y1}+{F}_{y2}\right)\mathrm{sin}\delta +{F}_{x3}+{F}_{x4},$$

2 $$m\left({\dot{V}}_{y}+r{V}_{x}\right)-{m}_{s}{h}_{s}\ddot{\mathrm{\varnothing }}=\left({F}_{y1}+{F}_{y2}\right)\mathrm{cos}\delta +\left({F}_{x1}+{F}_{x2}\right)\mathrm{sin}\delta +{F}_{y3}+{F}_{y4},$$

3 $${I}_{z}\dot{r}=\left({F}_{y1}+{F}_{y2}\right){l}_{f}\mathrm{cos}\delta +\left({F}_{y1}-{F}_{y2}\right)\frac{d}{2}\mathrm{sin}\delta -\left({F}_{y3}+{F}_{y4}\right){l}_{r}+\left({F}_{x1}+{F}_{x2}\right){l}_{f}\mathrm{sin}\delta -\left({F}_{x1}-{F}_{x2}\right)\frac{d}{2}\mathrm{cos}\delta +\left({F}_{x3}-{F}_{x4}\right)\frac{d}{2},$$

4 $${I}_{x}\ddot{\varnothing }={m}_{s}{h}_{s}\left({\dot{V}}_{y}+r{V}_{x}\right)+\left({m}_{s}{gh}_{s}-{K}_{\varnothing }\right)\varnothing -{C}_{\varnothing }\dot{\varnothing },$$

where $m$ is the mass of the vehicle; ${m}_{s}$ is the sprung mass; ${V}_{x}$ and ${V}_{y}$ are the longitudinal and lateral velocities; $r$ is the vehicle yaw rate; ${F}_{xi}$ and ${F}_{yi}$ are longitudinal and lateral forces of the four wheels ($i=1, 2, 3, 4$, representing the front left, front right, rear right and rear left wheels respectively); ${h}_{s}$ is the height from gravity center to the roll center; $\delta$ is the steering angle of the front wheels; $\varnothing$ is vehicle roll angle at the center of gravity; $g$ is the acceleration of gravity; ${I}_{z}$ and ${I}_{x}$ are the inertia moment about the vertical and longitudinal axis; ${l}_{f}$ and ${l}_{r}$ are the distances from the gravity center to the front and the rear axles respectively; $d$ is the vehicle track width; ${K}_{\varnothing }$ is the roll stiffness of the suspension; ${C}_{\varnothing }$ is the roll damping ratio of the suspension.

### Tire model

Since tires are the only connection between the vehicle and the ground, tire force is a real-time reflection of and may change the state of the vehicle. Therefore, the establishment of an accurate tire model is necessary for the dynamic simulation. In this study, tires are commonly in a nonlinear area when BYRIC is working, therefore, the magic tire model which can accurately describe the tire force in the nonlinear region is adopted and the corresponding expression is as follows^33–35^:

5 $$Y\left(X\right)=y\left(x\right)+{S}_{V},$$

6 $$y\left(x\right)=D\mathrm{sin}\left\{C\mathrm{arctan}\left[Bx-E\left(Bx-arctanBx\right)\right]\right\},$$

7 $$X=x+{S}_{H},$$

where $y\left(x\right)$ is the dependent variable and $x$ is the independent variable; $Y\left(X\right)$ represents longitudinal force, lateral force or aligning torque; $X$ represents longitudinal slip ratio or wheel sideslip angle; ${S}_{H}$ and ${S}_{V}$ are the horizontal and vertical drifts of the vehicle respectively; The stiffness factor $B$ tensile curve; The shape factor $C$ mainly affects the shape of the curve; The peak factor $D$ determines the peak value of the curve; The product $BCD$ corresponds to the slope of the curve at the origin. The curvature factor $E$ affects the curvature around the peak.

8 $$C=2-\frac{2}{\pi }arctan\frac{{y}_{\infty }}{D},$$

9 $$E=\frac{B{x}_{m}-tan\frac{\pi }{2C}}{B{x}_{m}-arctanB{x}_{m}},$$

where ${y}_{\infty }$ is the asymptotic value of output when $x$ is large, namely ${y}_{\infty }=D\left(sin\frac{\pi }{2}C\right)$; Peak position ${x}_{m}$ directly determines the curvature factor $E$. Under pure slip conditions, the longitudinal force for pure slip conditions (no slip angle) is:

10 $${F}_{x0}={D}_{x}\mathrm{sin}\left\{{C}_{x}\mathrm{arctan}\left[{B}_{x}\kappa -{E}_{x}\left({B}_{x}\kappa -arctan{B}_{x}\kappa \right)\right]\right\}.$$

The coefficients ${C}_{x}$, ${D}_{x}$ and ${E}_{x}$ are function of tire load ${F}_{z}$ and camber angle $\gamma$. The complete equation can be obtained from literatur^36^. The lateral force for pure slip (free rolling) is:

11 $${F}_{y0}={D}_{y}\mathrm{sin}\left\{{C}_{y}\mathrm{arctan}\left[{B}_{y}\alpha -{E}_{y}\left({B}_{y}\alpha -arctan{B}_{y}\alpha \right)\right]\right\}.$$

Under combined slip conditions (tire driving or braking while cornering), the force expression under combined slip condition is as follows:

12 $$\left\{\begin{array}{c}{F}_{x}={F}_{x0}\cdot {G}_{x\alpha }\\ {F}_{y}={F}_{y0}\cdot {G}_{y\kappa }\end{array}\right.,$$

where ${G}_{x\alpha }$ and ${G}_{y\kappa }$ are the weighting functions of longitudinal and lateral force respectively shown in Eqs. (13) and (14).

13 $${G}_{x\alpha }=\frac{cos\left\{{C}_{x\alpha }\mathrm{arctan}\left[{B}_{x\alpha }{\alpha }_{s}-{E}_{x\alpha }\left({B}_{x\alpha }{\alpha }_{s}-arctan{B}_{x\alpha }{\alpha }_{s}\right)\right]\right\}}{cos\left\{{C}_{x\alpha }\mathrm{arctan}\left[{B}_{x\alpha }{S}_{Hx\alpha }-{E}_{x\alpha }\left({B}_{x\alpha }{S}_{Hx\alpha }-arctan{B}_{x\alpha }{S}_{Hx\alpha }\right)\right]\right\}},$$

14 $${G}_{y\kappa }=\frac{cos\left\{{C}_{y\kappa }\mathrm{arctan}\left[{B}_{y\kappa }\kappa -{E}_{y\kappa }\left({B}_{y\kappa }\kappa -arctan{B}_{y\kappa }\kappa \right)\right]\right\}}{cos\left\{{C}_{y\kappa }\mathrm{arctan}\left[{B}_{y\kappa }{S}_{Hy\kappa }-{E}_{y\kappa }\left({B}_{y\kappa }{S}_{Hy\kappa }-arctan{B}_{y\kappa }{S}_{Hy\kappa }\right)\right]\right\}}.$$

The angular speed and slip ratio of wheels during driving of a vehicle can be expressed by Eqs. (15) and (16) respectively.

15 $${I}_{w}{\dot{\omega }}_{i}={T}_{di}-{T}_{bi}-{R}_{w}{F}_{xi},$$

16 $${\lambda }_{i}=\frac{{R}_{w}{\omega }_{i}-{V}_{wxi}}{\mathrm{max}\left({R}_{w}{\omega }_{i},{V}_{wxi}\right)},$$

where ${I}_{w}$ and ${R}_{w}$ are the moment inertia and effective tire radius of the wheel; ${\omega }_{i}$, ${\lambda }_{i}$, ${T}_{di}$, ${T}_{bi}$ and ${V}_{wxi}$ are the angular speed, slip ratio, driving torque, braking torque and center speed of the ${i}^{th}$ wheel. In this study, only braking conditions of a vehicle is explored, so ${T}_{di}=0$. The wheel center speed ${V}_{wxi}$ can be calculated by Eqs. (17), (18), (19) and (20).

17 $${V}_{wx1}=\left({V}_{x}-rT/2\right)\mathrm{cos}\delta +\left({V}_{y}+r{l}_{f}\right)\mathrm{sin}\delta ,$$

18 $${V}_{wx2}=\left({V}_{x}+rT/2\right)\mathrm{cos}\delta +\left({V}_{y}+r{l}_{f}\right)\mathrm{sin}\delta ,$$

19 $${V}_{wx3}={V}_{x}+rd/2,$$

20 $${V}_{wx4}={V}_{x}-rd/2,$$

The wheel slip angle can be calculated by Eqs. (21), (22).

21 $${\alpha }_{f}=\frac{{V}_{x}\beta +{l}_{f}r}{{V}_{x}}-\delta ,$$

22 $${\alpha }_{r}=\frac{{V}_{x}\beta -{l}_{f}r}{{V}_{x}},$$

where ${\alpha }_{f}$ and ${\alpha }_{r}$ are the front-wheel and rear-wheel slip angle respectively; $\beta$ is the sideslip angle.

Under the influence of longitudinal and lateral acceleration, the vertical load on each wheel will be transferred. The vertical load comprising static load and transferred load can be calculated by Eqs. (23), (24), (25) and (26).

23 $${F}_{Z1}=\frac{1}{2l}\left(mg{l}_{r}-h\sum {F}_{x}\right)-\frac{1}{d}\left({K}_{\varnothing f}\varnothing +{C}_{\varnothing f}\dot{\varnothing }+{h}_{f}\sum {F}_{yf}\right),$$

24 $${F}_{Z2}=\frac{1}{2l}\left(mg{l}_{r}-h\sum {F}_{x}\right)+\frac{1}{d}\left({K}_{\varnothing f}\varnothing +{C}_{\varnothing f}\dot{\varnothing }+{h}_{f}\sum {F}_{yf}\right),$$

25 $${F}_{Z3}=\frac{1}{2l}\left(mg{l}_{f}+h\sum {F}_{x}\right)+\frac{1}{d}\left({K}_{\varnothing r}\varnothing +{C}_{\varnothing r}\dot{\varnothing }+{h}_{r}\sum {F}_{yr}\right),$$

26 $${F}_{Z4}=\frac{1}{2l}\left(mg{l}_{f}+h\sum {F}_{x}\right)-\frac{1}{d}\left({K}_{\varnothing r}\varnothing +{C}_{\varnothing r}\dot{\varnothing }+{h}_{r}\sum {F}_{yr}\right),$$

where ${F}_{Zi}$ is the vertical load on the ${i}^{th}$ wheel; $l$ is the longitudinal wheel-base; $h$ is the height of gravity center; ${K}_{\varnothing f}$ and ${K}_{\varnothing r}$ are the roll stiffness of the front and rear suspension respectively; ${C}_{\varnothing f}$ and ${C}_{\varnothing r}$ are the roll damping ratio of the front and rear suspension respectively; ${h}_{f}$ and ${h}_{r}$ are the roll center heights of the front axle and rear axle respectively.

## Braking-yaw-roll integrated control strategy

**Figure 3:**
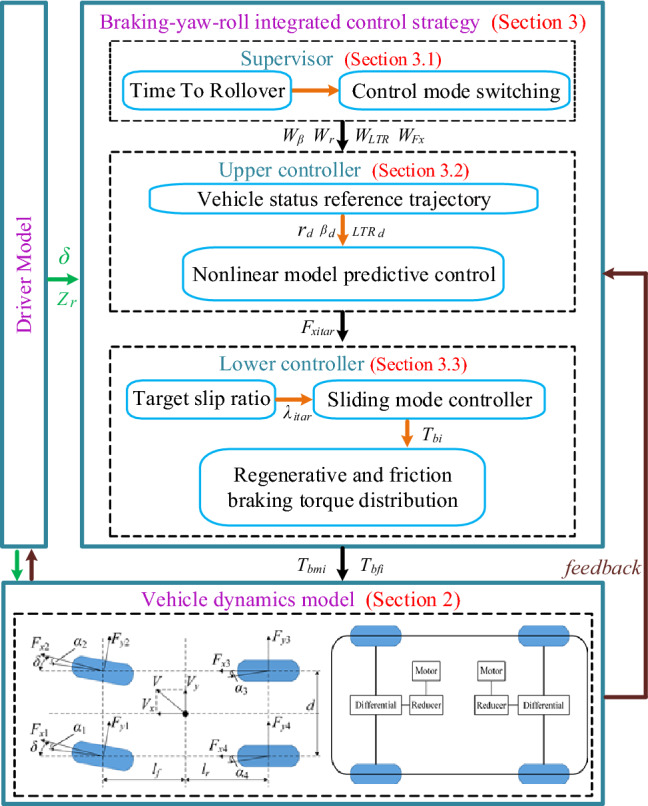
Framework of BYRIC.

Upon steering-braking at a high speed, a vehicle is likely to have the wheels locked, resulting in sharp decrease in the lateral tire force and further lateral instability of the vehicle. An SUV with a high gravity center, equipment with the conventional ABS and ESC can effectively prevent the wheels from locking and secure the lateral stability, but cannot secure the roll stability. To solve this problem, the BYRIC based on the dynamic index of rollover was proposed. The framework of BYRIC was shown in Fig. 3.

The control system consists of a supervisor, an upper controller and a lower controller. In the supervisor, a rollover prediction model is established to dynamically predict the vehicle rollover index. Vehicle control modes are divided into four types based on the rollover index and current vehicle states observed or collected by the sensors. The weight coefficients between yaw, roll and braking force are determined by the control mode to ensure that the BYRIC can work effectively in various conditions. The upper controller is a nonlinear model predictive control (NMPC) which is the core of the BYRIC. It takes the weight coefficients from supervisor and the reference vehicle status as inputs, and calculates the optimal distribution of the braking force ${F}_{xi}$ of the four wheels to ensure the yaw stability, roll stability and braking safety. Then it outputs the target tire longitudinal force ${F}_{xitar}$ to the lower controller. The lower controller converts the control of the tire longitudinal force into the control of the tire slip ratio to prevent wheel locking. Braking torque ${T}_{bi}$ of each wheel is calculated by sliding mode control and divided into two parts: regenerative braking and friction braking.

### Supervisor

#### Vehicle dynamic rollover index

In this study, load transfer ratio ($LTR$) and time to rollover ($TTR$) are used as the vehicle dynamic rollover indexes.

27 $$LTR=\left|\frac{{F}_{z1}+{F}_{z4}-{F}_{z2}-{F}_{z3}}{{F}_{z1}+{F}_{z2}+{F}_{z3}+{F}_{z4}}\right|.$$

Equation (27) is the theoretical calculation of $LTR$, representing the vertical load difference between the left and right sides of the vehicle. According to Eq. (27), $LTR$ ranges from 0 to 1. When $LTR=0$, it means that the vertical load on the left and right side are equal and the vehicle is of sound roll stability. When $LTR=1$, it means that the wheels on one side of the vehicle have already left or are about to leave the ground, exposing the vehicle to liable rollover. Due to interference of uncertain factors such as uneven road and lateral wind, the closer $LTR$ is to 1, the greater risk of rollover is posed by the interference. Therefore, it is required to keep $LTR$ at a low level. In this study, ${LTR}_{th}$ is set to 0.8 as the rollover threshold. When ${LTR>LTR}_{th}$, the vehicle is considered to be at risk of rollover.

Since the vertical load of each wheel can barely be measured in the actual braking conditions, it is impossible to calculate the $LTR$ of a vehicle in real-time by Eq. (27). Therefore, Eq. (8) is used to estimate the $LTR$.

28 $$LTR=\left|\frac{2\left({K}_{\varnothing }\varnothing +{C}_{\varnothing }\dot{\varnothing }\right)}{mgd}\right|.$$

**Figure 4:**
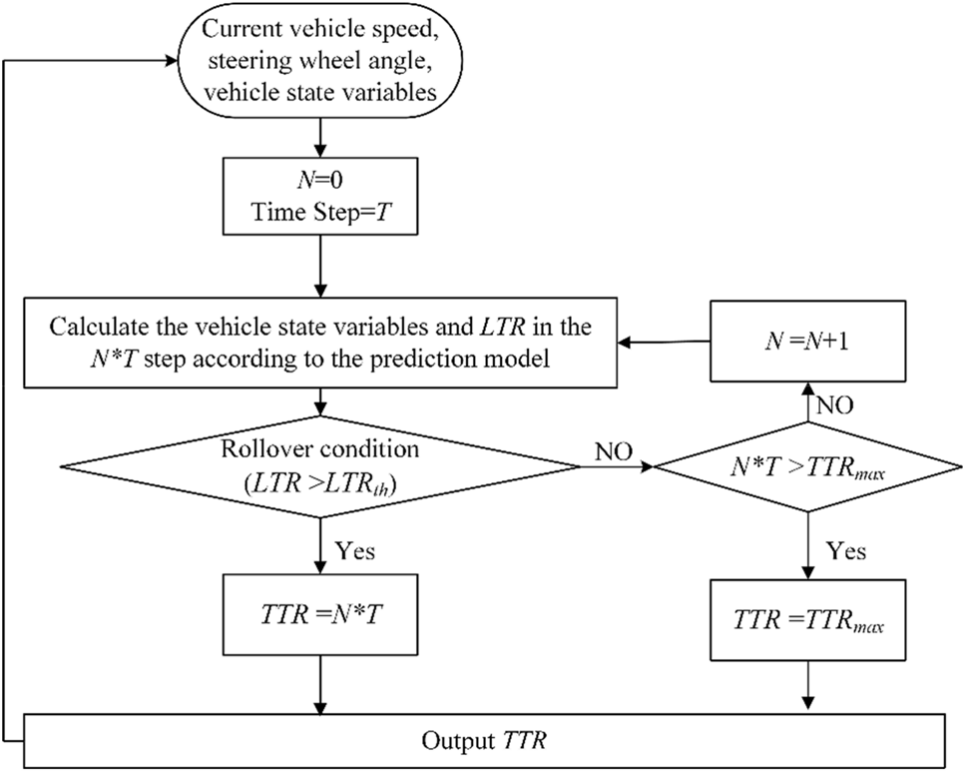
Calculation process of *TTR*.

Since there is a certain time delay in both the control system and the driver's operation, and it is hard to control the vehicle when the wheels on one side are about to leave the road ($LTR\ge {LTR}_{th}$), using $LTR$ as the only factor to determine whether the intervention of rollover control is needed cannot ensure that the vehicle rollover is effectively controlled. The $TTR$ was used as an index of rollover control intervention in this study to predict the time from the current state to the occurrence of rollover, so that the rollover prevention control system can intervene before the occurrence of rollover and spare enough time to maintain $LTR$ within a safe range. The calculation process of $TTR$ is shown in Fig. 4.

First, collect the current vehicle speed, steering angle and state variables used in the prediction model (${V}_{x},\beta ,r,\varnothing ,\dot{\varnothing }$); suppose the tire longitudinal force ${F}_{xi}$ remains constant in the process; use the prediction model to predict the value of $LTR$ after $N$ time steps $T$ and compare this value with the rollover threshold ${LTR}_{th}$. If the rollover condition is met $LTR\ge {LTR}_{th}$, then $TTR=N*T$. To avoid long-time cyclic calculation in case there is a low possibility of rollover under certain stable conditions, take ${TTR}_{max}$ as the upper limit of $TTR$, that is, when $N*T={TTR}_{max}$, terminate the cycle as the vehicle is considered as not exposed to the risk of rollover.

#### Control mode switching

According to vehicle states and the stability thresholds, the control of BYRIC can be divided into four modes: braking control, braking-yaw integrated control, braking-roll integrated control and braking-yaw-roll integrated control. The difference among the four control modes mainly lies in the different weight coefficients of the NMPC objective function $J$ which is defined in Eq. (34). The switching algorithm of the control mode is shown in Table 2. $\left|\Delta r\right|$ is the absolute value of the difference between the real yaw rate and the desired yaw rate. When $\left|\Delta r\right|>\Delta {r}_{th}$, it means that there is a risk of sideslip and the intervention of yaw control is required. When $TTR<{TTR}_{th}$, it means that there is a risk of rollover and the intervention of rollover prevention control is required. ${W}_{\beta }$, ${W}_{r}$, ${W}_{LTR}$ and ${W}_{Fx}$ are the weight coefficients required by the upper controller. The four coefficients of each mode are derived from a series of experiments and evaluations.

**Table 2:** Switching algorithm of the control mode.

Vehicle states	Control model	$\left[{W}_{\beta },{W}_{r},{W}_{LTR},{W}_{Fx}\right]$
$\left|\Delta r\right|<\Delta {r}_{th},TTR\ge {TTR}_{th}$	Braking	$\left[0, 0, 0, 0.05\right]$
$\left|\Delta r\right|>\Delta {r}_{th},TTR\ge {TTR}_{th}$	Braking-yaw	$\left[\mathrm{0.02,80,0.05}\right]$
$\left|\Delta r\right|<\Delta {r}_{th},TTR<{TTR}_{th}$	Braking-roll	$\left[0, 80, 0, 0.05\right]$
$\left|\Delta r\right|>\Delta {r}_{th},TTR<{TTR}_{th}$	Braking-yaw-roll	$\left[0.02, 80, 80, 0.05\right]$

### Upper controller

#### Vehicle status reference trajectory

NMPC is used in the upper controller to achieve the control objectives of braking safety, yaw stability and roll stability. To achieve an ideal control effect, it is important to set a reasonable reference trajectory for the yaw rate, sideslip angle and $LTR$.

**Figure 5:**
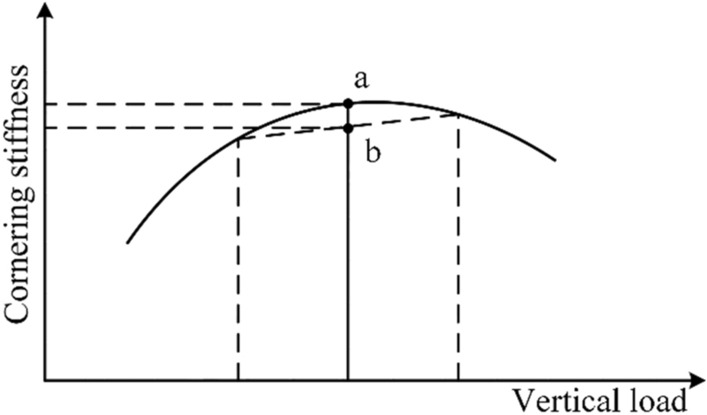
Relationship between cornering stiffness and vertical load.

The reference yaw rate ${r}_{d}$ and sideslip angle ${\beta }_{d}$ can be obtained as follows^37,38^:

29 $${r}_{d}=\mathrm{min}\left\{\left|\frac{{V}_{x}/l}{1+K{V}_{x}^{2}}\delta \right|,\left|\frac{\mu g}{{V}_{x}}\right|\right\}\cdot \mathrm{sgn}\left(\delta \right),$$

30 $${\beta }_{d}=\mathrm{min}\left\{\left|\frac{\delta }{l\left(1+K{V}_{x}^{2}\right)}\left({l}_{r}-\frac{m{l}_{f}{V}_{x}^{2}}{l{K}_{r}}\right)\right|,\left|\mathrm{arctan}\left(0.02\mu g\right)\right|\right\}\cdot \mathrm{sgn}\left(\delta \right),$$

where $K$ is the stability factor of the vehicle. For $LTR$, the larger the $LTR$ is, the greater the risk of rollover caused by external interference or sprung mass roll inertia is. In addition, it can be seen from the relationship between cornering stiffness and vertical load in Fig. 5 that load transfer will reduce the average cornering stiffness of the tire and then weaken the lateral stability of the vehicle. Therefore, ${LTR}_{d}=0$ is adopted as the desired $LTR$.

#### Nonlinear model predictive control

Considering that: (1) vehicle dynamics is a complex nonlinear system; (2) braking safety, yaw stability and roll stability need to be achieved simultaneously; (3) the variables need to be constrained during the process, NMPC is the most appropriate control method.

##### Establishment of the prediction model

Combining Eqs. (1), (2), (3), (4) and (5), take the longitudinal speed ${V}_{x}$, sideslip angle $\beta$, yaw rate $r$, roll angle $\varnothing$, the differential of roll angle $\dot{\varnothing }$ as state variables. Take the vehicle sideslip angle $\beta$, yaw rate $r$ and load transfer ratio $LTR$ as the output variables. The vehicle dynamics state-space model can be expressed as:

31 $$\dot{{\varvec{x}}}=f\left({\varvec{x}},{\varvec{u}}\right),$$

32 $${\varvec{y}}=C{\varvec{x}},$$

where $${\varvec{x}}={[{V}_{x} \beta r\mathrm{ \varnothing }\dot{\mathrm{\varnothing }}]}^{T}$$, $${\varvec{u}}={\left[{F}_{x1} {F}_{x2} {F}_{x3} {F}_{x4}\right]}^{T}$$, $${\varvec{y}}={\left[\beta r LTR\right]}^{T}$$,$$C=\left[\begin{array}{ccccc}0& 1& 0& 0& 0\\ 0& 0& 1& 0& 0\\ 0& 0& 0& \frac{2{K}_{\mathrm{\varnothing }}}{mgd}& \frac{2{C}_{\mathrm{\varnothing }}}{mgd}\end{array}\right]$$.

To simplify the calculation and ensure real-time control, it is assumed that the tire lateral force works in the linear region, namely: ${F}_{yf}={F}_{y1}+{F}_{y2}={K}_{f}{\alpha }_{f}$, ${F}_{yr}={F}_{y3}+{F}_{y4}={K}_{r}{\alpha }_{r}$, where, ${K}_{f}$ and ${K}_{r}$ are the cornering stiffness of the front axle and rear axle respectively. The vehicle state model is rewritten as follows:

33 $$\dot{{\varvec{x}}}=\left[\begin{array}{c}\frac{1}{m}\left[\left({F}_{x1}+{F}_{x2}\right)\mathrm{cos}\delta -{K}_{f}\left(\frac{{V}_{x}\beta +{rl}_{f}}{{V}_{x}}-\delta \right)\mathrm{sin}\delta +{F}_{x3}+{F}_{x4}-{m}_{s}{h}_{s}\dot{\varnothing }r+m{V}_{x}\beta r\right]\\ \frac{1}{m{V}_{x}}\left[{K}_{f}\left(\frac{{V}_{x}\beta +{rl}_{f}}{{V}_{x}}-\delta \right)\mathrm{cos}\delta +\left({F}_{x1}+{F}_{x2}\right)\mathrm{sin}\delta +{K}_{f}\left(\frac{{V}_{x}\beta -{rl}_{f}}{{V}_{x}}\right)-r-\frac{\beta }{{V}_{x}}{\dot{V}}_{x}\right]\\ \frac{1}{{I}_{z}}\left[\begin{array}{c}{K}_{f}{l}_{f}\left(\frac{{V}_{x}\beta +{rl}_{f}}{{V}_{x}}-\delta \right)cos\delta -{K}_{r}{l}_{r}\left(\frac{{V}_{x}\beta -{rl}_{f}}{{V}_{x}}\right)+\left({F}_{x1}+{F}_{x2}\right){l}_{f}sin\delta \\ -\left({F}_{x1}-{F}_{x2}\right)\frac{d}{2}cos\delta +\left({F}_{x3}-{F}_{x4}\right)\frac{d}{2}\end{array}\right]\\ \dot{\varnothing }\\ \frac{1}{{I}_{x}}\left\{\begin{array}{c}\frac{{m}_{s}{h}_{s}}{m}\left[{K}_{f}\left(\frac{{V}_{x}\beta +{rl}_{f}}{{V}_{x}}-\delta \right)\mathrm{cos}\delta +\left({F}_{x1}+{F}_{x2}\right)\mathrm{sin}\delta +{K}_{f}\left(\frac{{V}_{x}\beta -{rl}_{f}}{{V}_{x}}\right)\right]\\ +\left({m}_{s}{gh}_{s}-{K}_{\varnothing }\right)\varnothing -{C}_{\varnothing }\dot{\varnothing }\end{array}\right\}\end{array}\right].$$

##### Design of the objective function

To achieve the desired yaw rate, sideslip angle and $LTR$, as well as follow the desired braking intensity, the objective function of the NMPC controller is defined as follows:

34 $$\underset{{u}_{i}}{\mathrm{min}}J={W}_{\beta }{\Vert {Y}_{1}\left(k+1|k\right)-{\beta }_{d}\left(k+1\right)\Vert }^{2}+{W}_{r}{\Vert {Y}_{2}\left(k+1|k\right)-{r}_{d}\left(k+1\right)\Vert }^{2}+{W}_{LTR}{\Vert {Y}_{3}\left(k+1|k\right)\Vert }^{2}-\uppsi {W}_{Fx}{\sum }_{i=1}^{4}{\Vert {u}_{i}\left(k\right)\Vert }^{2}+{\sum }_{i=1}^{4}{\Vert {\Delta u}_{i}\left(k\right)\Vert }^{2},$$

where ${W}_{\beta }$, ${W}_{r}$, ${W}_{LTR}$ and ${W}_{Fx}$ are the weight coefficients of $\beta$, $r$, $LTR and$ the braking force; $\uppsi$ is the braking force switching factor; ${Y}_{1}\left(k+1|k\right)$, ${Y}_{2}\left(k+1|k\right)$ and ${Y}_{3}\left(k+1|k\right)$ are the prediction output sequences of $\beta$, $r$ and $LTR$ at step $k$; ${\beta }_{d}\left(k+1\right)$ and ${r}_{d}\left(k+1\right)$ are the desired input sequences of $\beta$ and $r$; ${u}_{i}\left(k\right)$ is the optimal control input sequence of the ${i}^{th}$ wheel; and ${\Delta u}_{i}\left(k\right)$ is the optimal control input increment sequence of the ${i}^{th}$ wheel. Among them,

$${Y}_{1}\left(k+1\left|k\right.\right)=\left[\begin{array}{c}\beta \left(k+1\left|k\right.\right)\\ \beta \left(k+2\left|k\right.\right)\\ \vdots \\ \beta \left(k+p\left|k\right.\right)\end{array}\right]{ Y}_{2}\left(k+1\left|k\right.\right)=\left[\begin{array}{c}r\left(k+1\left|k\right.\right)\\ r\left(k+2\left|k\right.\right)\\ \vdots \\ r\left(k+p\left|k\right.\right)\end{array}\right] {Y}_{3}\left(k+1\left|k\right.\right)=\left[\begin{array}{c}LTR\left(k+1\left|k\right.\right)\\ LTR\left(k+2\left|k\right.\right)\\ \vdots \\ LTR\left(k+p\left|k\right.\right)\end{array}\right],$$

$${\beta }_{d}\left(k+1\right)=\left[\begin{array}{c}{\beta }_{d}\left(k+1\left|k\right.\right)\\ {\beta }_{d}\left(k+2\left|k\right.\right)\\ \vdots \\ {\beta }_{d}\left(k+p\left|k\right.\right)\end{array}\right] {r}_{d}\left(k+1\right)=\left[\begin{array}{c}{r}_{d}\left(k+1\left|k\right.\right)\\ {r}_{d}\left(k+2\left|k\right.\right)\\ \vdots \\ {r}_{d}\left(k+p\left|k\right.\right)\end{array}\right],$$

$${u}_{i}\left(k\right)=\left[\begin{array}{c}{u}_{i}\left(k\left|k\right.\right)\\ {u}_{i}\left(k+1\left|k\right.\right)\\ \vdots \\ {u}_{i}\left(k+m-1\left|k\right.\right)\end{array}\right] {\Delta u}_{i}\left(k\right)=\left[\begin{array}{c}\Delta {u}_{i}\left(k+1\left|k\right.\right)\\ \Delta {u}_{i}\left(k+2\left|k\right.\right)\\ \vdots \\ {\Delta u}_{i}\left(k+m-1\left|k\right.\right)\end{array}\right].$$

The calculation of the braking force switching factor $\uppsi$ is as follows: The maximum braking force $\left|\sum_{i=1}^{4}u\left(i\right)\right|$ is calculated on the premise of secured yaw and roll stability based on the current driver’s desired braking intensity, steering angle and state variables, and is compared with the driver’s desired braking force $mg{z}_{r}$, where ${z}_{r}$ is the driver's desired braking intensity which is greater than or equal to 0 and ${z}_{r}=0$ means the driver expects to drive at a constant speed. If $\left|\sum_{i=1}^{4}u\left(i\right)\right|>mg{z}_{r}$, it means that the maximum tire braking force meets the driver's braking intention and the braking force switching factor $\uppsi =0$. If $\left|\sum_{i=1}^{4}u\left(i\right)\right|<mg{z}_{r}$, the maximum tire braking force does not meet the driver’s braking intention, in this case, $\uppsi =1$.

##### Setting of constraints

In this study, the longitudinal force of the four wheels is taken as the control input. The longitudinal force cannot be directly controlled, but can be controlled by applying braking torque to the wheels. Therefore, considering the braking capacity, the torque output capacity of the motor and the road adhesion conditions, the control input should meet the following constraints:

35 $${u}_{min}={\left[{F}_{x1min} {F}_{x2min} {F}_{x3min} {F}_{x4min}\right]}^{T},$$

36 $${u}_{max}={\left[0 0 0 0\right]}^{T},$$

37 $$\Delta {u}_{min}={\left[{\Delta F}_{x1min} {\Delta F}_{x2min} {\Delta F}_{x3min} {\Delta F}_{x4min}\right]}^{T},$$

38 $$\Delta {u}_{max}={\left[{\Delta F}_{x1max} {\Delta F}_{x2max} {\Delta F}_{x3max} {\Delta F}_{x4max}\right]}^{T},$$

39 $${u}_{min}\le u\le {u}_{max,}$$

40 $$\Delta {u}_{min}\le \Delta u\le \Delta {u}_{max},$$

where ${u}_{min}$ and ${u}_{max}$ are the minimum and maximum braking forces respectively. $\Delta {u}_{min}$ and $\Delta {u}_{max}$ are the minimum and maximum increments of braking force respectively. ${F}_{ximin}(i=1, 2, 3, 4)$ is the maximum longitudinal tire force which is defined in Eq. (38). ${\Delta F}_{ximin}$ and ${\Delta F}_{ximax}$ are the minimum and maximum increments of the braking force in a prediction step ${T}_{p}$, mainly subject to the response speed of the braking system.

Considering braking safety and according to the Economic Commission for Europe (ECE) braking regulations, the utilization adhesion coefficient curve of the front axle shall be above that of the rear axle under various loading conditions. However, if the utilization adhesion coefficient curve of the rear axle does not go beyond line $z+0.05$ when the braking intensity is between 0.3 and 0.45, the utilization adhesion coefficient curve of the rear axle could be above that of the front axle, so that the constraints of control input $u$ can be obtained as:

41 $$\left\{\begin{array}{c}\left(\frac{1}{\left({l}_{r}+zh\right)}\left|{F}_{xf}\right|<\frac{1}{\left({l}_{f}-zh\right)}\left|{F}_{xr}\right|\right)\bigcap \left(\left|{F}_{xf}\right|\le \frac{z+0.07}{0.85}\cdot \frac{mg\left({l}_{r}+zh\right)}{L}\right) \left(z<0.3\right)\bigcup \left(z>0.45\right)\\ \left(\frac{1}{mg\left({l}_{f}-zh\right)}\left|{F}_{xr}\right|<z+0.05\right)\bigcap \left(\left|{F}_{xf}\right|\le \frac{z+0.07}{0.85}\cdot \frac{mg\left({l}_{r}+zh\right)}{L}\right) 0.3<z<0.45\end{array}\right.,$$

where $z$ is the braking intensity. In addition, to make the actual braking intensity follow the driver's braking intention, the control input should also meet the following constraint:

42 $$\sum {F}_{xi}=-mg{z}_{r}$$

Under the conditions of high braking intensity, low road adhesion coefficient, or significant vehicle yaw rate where the maximum tire longitudinal force is constrained by both the road adhesion coefficient and the adhesion ellipse, the vehicle cannot achieve the desired braking intensity. In this scenario, the control aims to make the actual braking intensity as close to the desired braking intensity as possible by maximizing the braking force on the premise of secured yaw and roll stability. For further details on the asymptotic stability of NMPC, please refer to Appendix A.

### Lower controller

The lower controller functions to achieve the target tire longitudinal force ${F}_{xitar}$ from the upper controller by controlling the braking torque ${T}_{bi}$. To prevent wheel locking, it converts the control of the tire longitudinal force into the control of the tire slip ratio.

#### Target slip ratio

**Figure 6:**
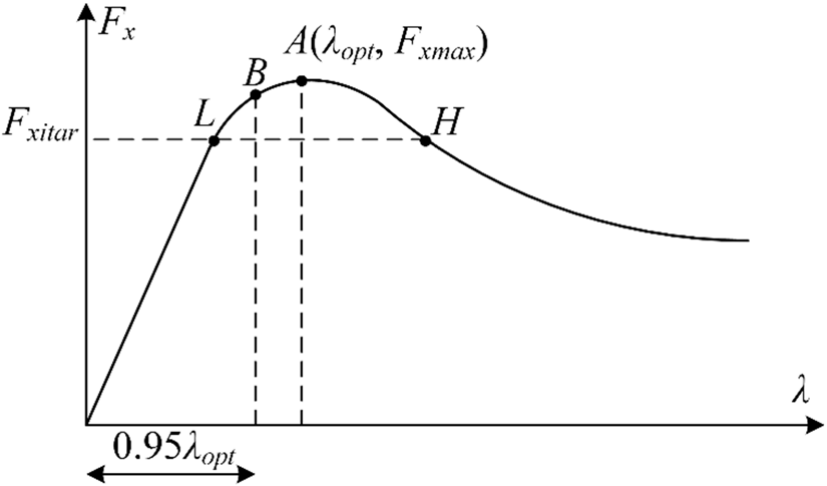
Relationship between tire longitudinal force and tire slip ratio.

According to the magic formula tire model Eqs. (5), (6) and (7), the tire longitudinal force ${F}_{xi}$ is a quaternary function of vertical load ${F}_{zi}$, tire slip angle ${\alpha }_{i}$, tire slip ratio ${\lambda }_{i}$ and road adhesion coefficient $\mu$ (Fig. 6), and is represented as follows:

43 $${F}_{xi}=h\left({F}_{zi },{\alpha }_{i},{\lambda }_{i},\mu \right).$$

If the current ${F}_{zi}$, ${\alpha }_{i}$ and $\mu$ are known, then ${F}_{xi}$ can be regarded as a univariate function of ${\lambda }_{i}$ under the current vehicle state. Figure 6 shows a diagram of the relationship between tire longitudinal force ${F}_{x}$ and tire slip ratio $\lambda$, according to which the maximum tire longitudinal force ${F}_{xmax}$ occurs at point A.

44 $${F}_{xmax}={h\left({F}_{zi },{\alpha }_{i},{\lambda }_{i},\mu \right)|}_{\frac{\partial h}{\partial \lambda }=0}.$$

When the tire slip ratio passes point A, the braking force coefficient starts to decrease and the lateral force coefficient drops sharply. Generally speaking, a certain target longitudinal force ${F}_{xitar}$ of each wheel corresponds to two slip ratios, ${\lambda }_{L}$ and ${\lambda }_{H}$. At point H, the tire is in a non-linear region which is relatively uncontrollable. So the lower slip ratio at point L is adopted as the target slip ratio corresponding to the target tire longitudinal force ${F}_{xitar}$.

45 $${\lambda }_{itar}=\mathrm{min}\left({h}^{-1}\left({F}_{xitar}\right)\right).$$

#### Sliding mode controller

In this study, sliding mode control is used to track the target slip ratio due to its strong robustness, fast response, and ability to handle nonlinear problems and suppress chattering. The sliding mode surface is defined as follow:

46 $$s={\lambda }_{itar}-{\lambda }_{i}.$$

According to the exponential reaching law,

47 $$\dot{s}=-\varepsilon \mathrm{sgn}\left(s\right)-{k}_{d}s,$$

where $\varepsilon$ and ${k}_{d}$ are the reaching law parameters, $\varepsilon >0$, ${k}_{d}>0$. The stability analysis of the sliding mode controller is shown in Appendix B.

Combining Eqs. (15), (16), (46) and (47), the control law of the braking torque can be obtained as:

48 $${T}_{bi}=-\frac{{I}_{w}{V}_{wxi}}{R}\left(\frac{1+{\lambda }_{i}}{{V}_{wxi}}{\dot{V}}_{wxi}+\frac{{R}^{2}{F}_{xi}}{{I}_{w}{V}_{wxi}}+{\dot{\lambda }}_{itar}+\varepsilon \mathrm{sgn}\left(s\right)+{k}_{d}s\right).$$

**Figure 7:**
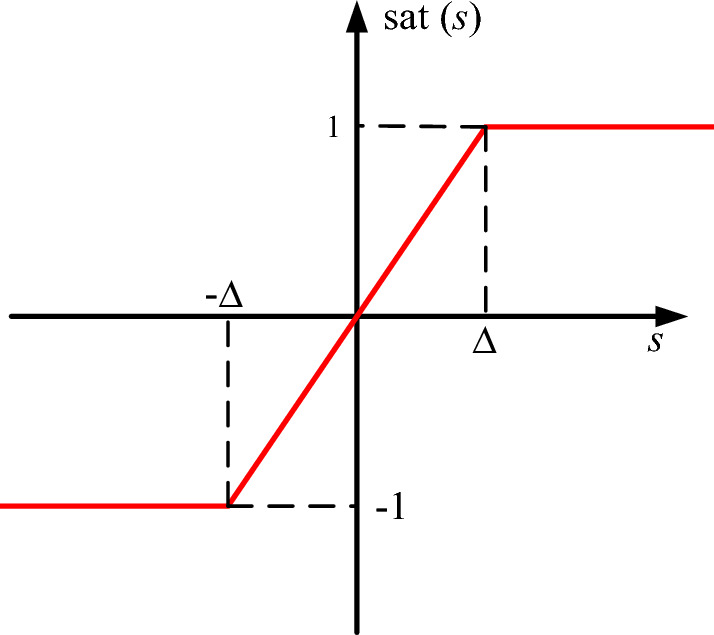
Diagram of saturation function.

To suppress the chattering of sliding mode control, the sign function $\mathrm{sgn}(s)$ is replaced by the saturation function $\mathrm{sat}(s)$. The expression of $\mathrm{sat}(s)$ is given by Eq. (49) and its schematic diagram is shown in Fig. 7.

49 $$\mathrm{sat}\left(s\right)=\left\{\begin{array}{c}1, s>\Delta \\ \frac{s}{\Delta }, \left|s\right|\le 0\\ -1, s<-\Delta \end{array},\right.$$

where $\Delta$ is the thickness of boundary layer.

#### Regenerative braking torque and friction braking torque distribution

Compared with friction braking, motor regenerative braking is advantageous for rapid response and high control accuracy^39^, and can recover partial braking energy to extend the driving range. Nevertheless, the maximum braking torque provided by the motor is limited. To solve this problem, a regenerative-friction hybrid braking strategy is proposed, where motor regenerative braking is preferentially adopted and any excessive braking torque is compensated by friction braking.

**Figure 8:**
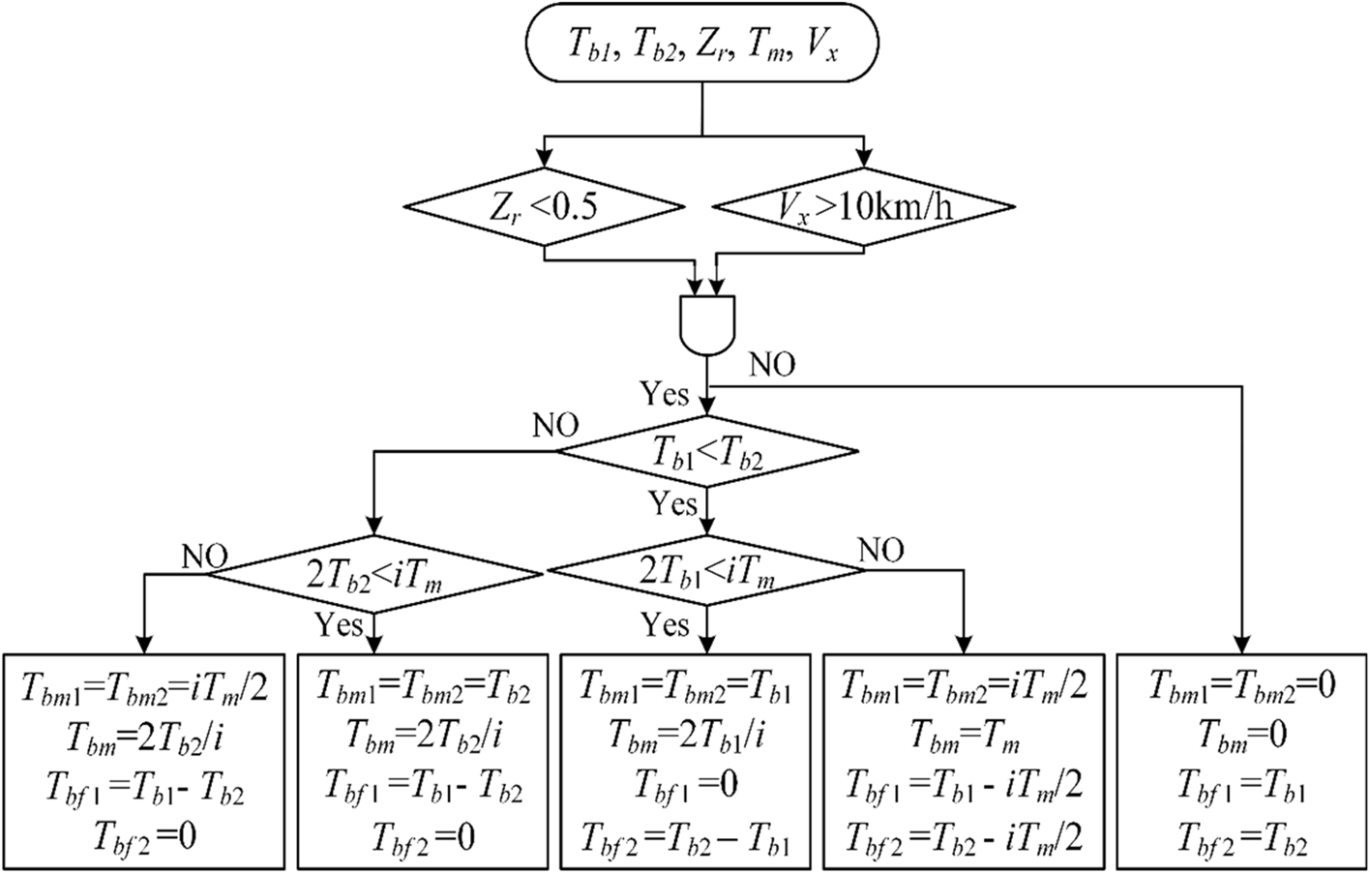
Regenerative-friction braking torque distribution strategy on the front axle.

A vehicle should be considered as being in an emergency braking when the driver's braking intention exceeds 0.5. To ensure braking safety and reliability in this case, regenerative braking will exit^40^. When the vehicle speed drops to 10 km/h, regenerative braking does not work, which means that the required braking torque is completely provided by friction braking. Take the wheels on the left and right sides of the front axle as an example, the regenerative-friction braking torque distribution strategy is shown in Fig. 8.

First, determine whether regenerative braking is involved based on the driver's braking intention and vehicle speed. If regenerative braking is involved, according to the structure of the FRID-EV shown in Fig. 1, it is deemed that regenerative braking torque distribution of the differential to the left and right half shafts is equal since the internal friction torque of the differential is small. Suppose the target braking torque ${T}_{b1}$ of the left wheel on the front axle is smaller than the target braking torque ${T}_{b2}$ of the right wheel on the front axle. Compare the smaller target braking torque ${T}_{b1}$ with the maximum regenerative braking torque $i{T}_{m}/2$ provided by the motor to the wheels at the current motor speed ($i$ is the transmission ratio of the reducer). If ${T}_{b1}<i{T}_{m}/2$, the left wheel braking torque ${T}_{b1}$ is all provided by motor regenerative braking and the right wheel braking torque ${T}_{b2}$ is provided by regenerative-friction hybrid braking in which the regenerative braking torque ${T}_{bm2}$ equals to the left wheel regenerative braking torque ${T}_{bm1}$ and the rest torque is compensated by friction braking, that is ${T}_{bf2}={T}_{b2}-{T}_{bm2}$. If ${T}_{b1}>i{T}_{m}/2$, it means that the regenerative braking torque of the motor cannot meet the braking torque requirement of either the left or the right wheel, the regenerative braking torque of the left and right wheels is the maximum regenerative braking torque that the motor can provide and the rest braking torque is provided by friction braking. The regenerative/friction braking torque of the wheels on the rear axle can be calculated similarly.

## Simulation results

**Figure 9:**
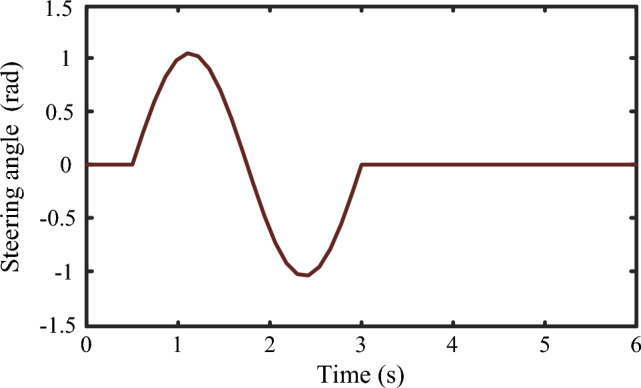
Simulation steering input.

To verify the performance of the proposed BYRIC under steering-braking condition, simulation experiments were carried out on the MATLAB/Simulink platform. The BYRIC was tested under the conditions of high and low road adhesion coefficients, corresponding to good and bad road conditions. The simulation steering input was shown in Fig. 9:

### High-adhesion coefficient road

**Figure 10:**
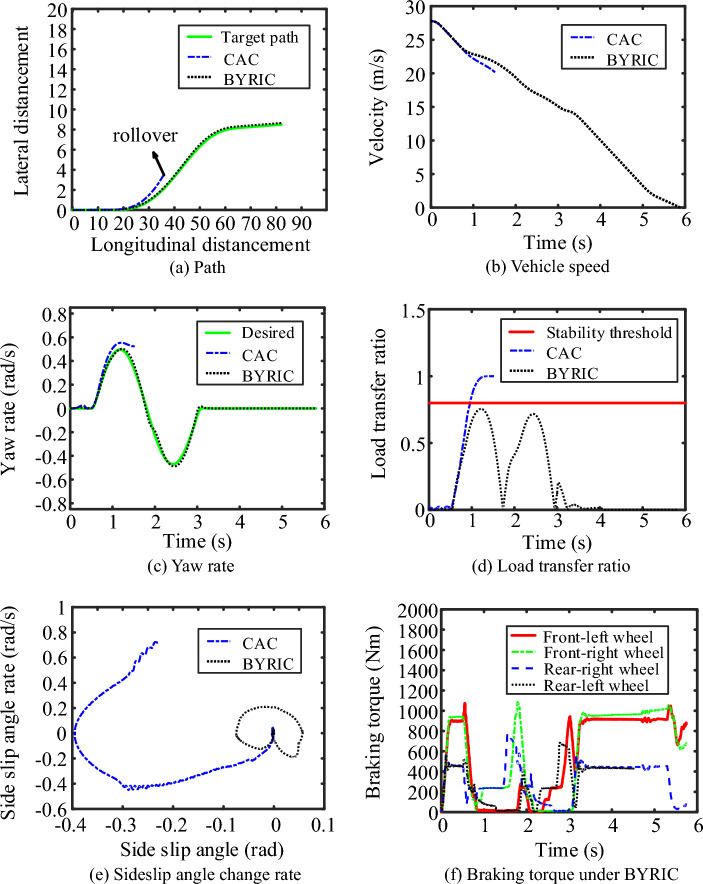
Simulation results under the condition of high-adhesion coefficient road.

The single lane change maneuver of Fig. 9 is used to verify the performance of BYRIC under the condition of steering-braking on a high-adhesion coefficient road. The initial speed of the vehicle was set to 100 km/h, the road adhesion coefficient $\mu =0.8$ and the driver's braking intention is 0.7. The vehicle starts to brake from $t=0$. For comparison, simulations of BYRIC and CAC were performed in this study. The simulation results are shown in Fig. 10.

It can be seen from Fig. 10a and b that before 0.7 s when the longitudinal displacement is 20 m and the steering angle is zero, BYRIC and the CAC have the same control effect on the vehicle and both can make the braking intensity of the vehicle follow the driver's braking intention; after 0.7 s, as the steering angle continues to increase, the vehicle under the control of CAC begins to sideslip and the driving path obviously deviates from the target path; rollover occurs at the longitudinal displacement of 36 m. In contrast, as can be seen from Fig. 10a,c and d, BYRIC can make the vehicle better follow the desired yaw rate and the target driving path, while reducing the vehicle’s load transfer ratio to avoid rollover due to an excessive load transfer ratio. Apart from the yaw rate and driving path, the vehicle’s lateral stability is also reflected in the phase plan of the sideslip angle and the sideslip angle change rate. As shown in Fig. 10e, the vehicle sideslip angle under BYRIC converges to zero faster than that under CAC. The braking torque is shown in Fig. 10f. Under BYRIC, regenerative braking exits and the braking torque are all provided by friction braking upon emergency braking (when the driver’s braking intention is set to 0.7).

### Low-adhesion coefficient road

**Figure 11:**
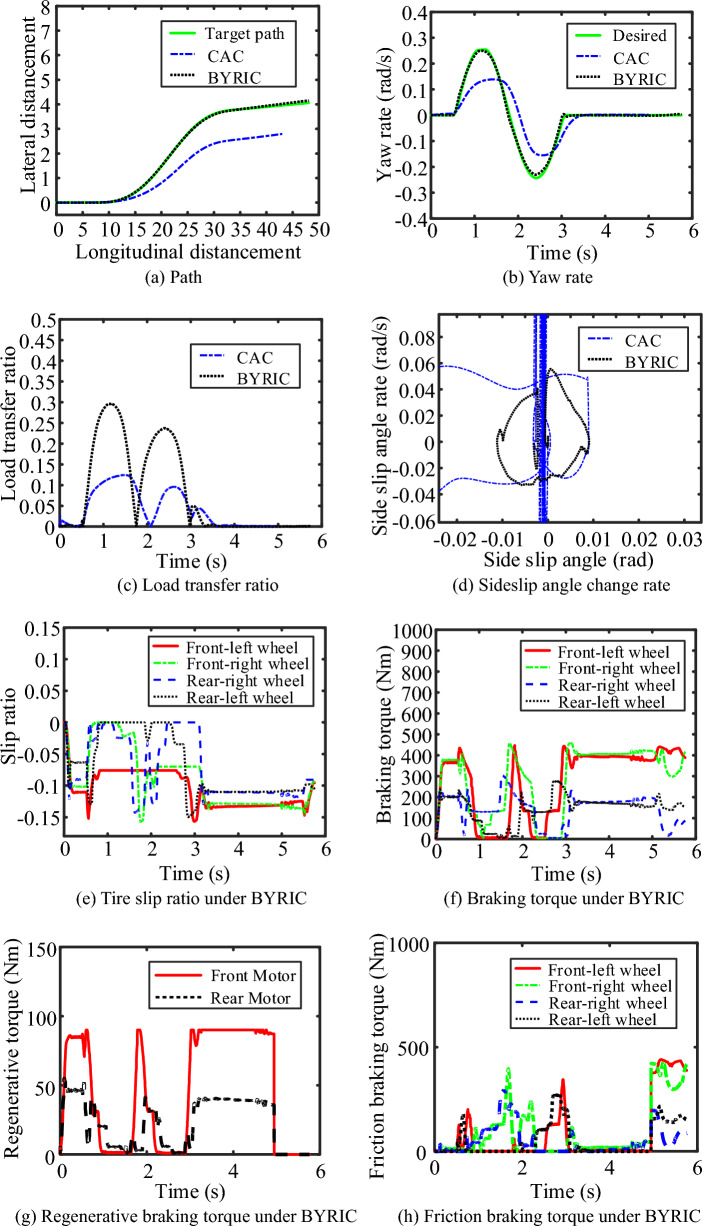
Simulation results under the condition of low-adhesion coefficient road.

To verify the performance of BYRIC under the condition of steering-braking on a low-adhesion coefficient road, the initial speed of the vehicle was set to 50 km/h, the road adhesion coefficient $\mu =0.3$ and the driver’s braking intention was set 0.4. The simulation results of BYRIC and CAC were shown in Fig. 11.

It can be seen from Fig. 11a and b, in a low road adhesion coefficient, the CAC can only realize approximation of the tire longitudinal force to the maximum value other than the lateral force, resulting in a much lower yaw rate than the desired yaw rate and eventual deviation of the vehicle from the target path. BYRIC can realize a greater lateral force of tires, rendering a higher LTR than that under the CAC in Fig. 11c, but both are within the safe range. It can be seen from Fig. 11d the phase trajectory range under BYRIC is smaller, which means that the vehicle is more stable. BYRIC can effectively control the tire slip ratio within an appropriate range to obtain a greater braking force (Fig. 11e). Besides, BYRIC can also recover partial braking energy with secured braking safety, yaw stability and roll stability due to the intervention of regenerative braking. The total braking torque, regenerative braking torque and the friction braking torque of the four wheels under BYRIC are respectively shown in Fig. 11f–h. In conclusion, BYRIC outperforms the CAC in terms of comprehensive active safety performance in a low adhesion road.

## Conclusion

In this study, a front and rear axle independent drive electric SUV was taken as the research object. According to the characteristics of the independent and controllable four-wheel braking of FRID-EV, based on the influence of different four-wheel braking force on braking safety, yaw stability and roll stability, the BYRIC strategy based NMPC was proposed, which considered the braking intention of the driver. The proposed BYRIC was compared with conventional ABS under steering-braking conditions and on different adhesion coefficient roads. The simulation results show that the BYRIC controller can effectively prevent the vehicle from rollover as well as accurately track the ideal path, yaw rate, sideslip angle and LTR which means better braking safety, yaw stability and roll stability under complex steering and braking conditions. In addition, the controller can effectively allocate the proportion of regenerative braking torque and friction braking torque during the process. However, since daily driving behavior includes a large number of steering-braking conditions, economy is also a factor worth further research. Besides, real vehicle verification can be considered to further verify the reliability of the algorithm. These results provide targeted directions for future research.

### Supplementary Information

Supplementary Information.

## Data Availability

The datasets used and/or analyzed during the current study available from the corresponding author on reasonable request.

## Abbreviations

ABS: Anti-lock braking system
ESP: Electronic stability program
DYC: Direct yaw moment control
AFS: Active front wheel steering system
ARS: Active rear wheel steering system
SUV: Sport utility vehicle
PLTR: Predictive lateral load transfer ratio
LTR: Load transfer ratio
MPC: Model predictive control
BYRIC: Braking-yaw-roll integrated control
FRID-EV: Front and rear axle independent drive electric vehicle
NMPC: Nonlinear model predictive control
ECE: The Economic Commission for Europe
CAC: Conventional ABS control

## Symbols

$$m$$: Vehicle mass
$$d$$: Wheel-track
${l}_{f}$: Distance between front axle and center of gravity
${l}_{r}$: Distance between rear axle and center of gravity
${T}_{max}$: Motor peak torque
${P}_{max}$: Motor peak power
${n}_{e}$: Motor rated speed
$${LTR}_{th}$$: Rollover threshold
$\varepsilon$: Reaching law parameter
${k}_{d}$: Reaching law parameter
$${m}_{s}$$: Sprung mass
$${V}_{x}$$: Longitudinal velocity
$${V}_{y}$$: Lateral velocity
$$r$$: Yaw rate
*i*: $i=1, 2, 3, 4$, representing the front left, front right, rear right and rear left wheels respectively
${F}_{xi}$: Longitudinal force of the ${i}^{th}$ wheel
${F}_{yi}$: Lateral force of the ${i}^{th}$ wheel
${h}_{s}$: The height from gravity center to the roll center
$$\delta $$: Steering angle
$$\varnothing $$: Roll angle
$g$: Acceleration of gravity
${I}_{z}$: Inertia moment about the vertical axis
${I}_{x}$: Inertia moment about the longitudinal axis
${l}_{f}$: Distances from the gravity center to the front axle
${l}_{r}$: Distances from the gravity center to the rear axle
${K}_{\varnothing }$: Roll stiffness of the suspension
${C}_{\varnothing }$: Roll damping ratio of the suspension
${S}_{H}$: The horizontal drifts
${S}_{V}$: The vertical drifts
$$B$$: Stiffness factor
$$C$$: Shape factor
$$D$$: Peak factor
$$E$$: Curvature factor
$$\pi $$: PI
$$\gamma $$: Camber angle
$${I}_{w}$$: Moment inertia
${R}_{w}$: Effective tire radius of the wheel
${\omega }_{i}$: The angular speed of the ${i}^{th}$ wheel
${\lambda }_{i}$: Slip ratio of the ${i}^{th}$ wheel
${T}_{di}$: Driving torque of the ${i}^{th}$ wheel
${T}_{bi}$: Braking torque of the ${i}^{th}$ wheel
${V}_{wxi}$: Center speed of the ${i}^{th}$ wheel
${\alpha }_{f}$: Front-wheel slip angle
${\alpha }_{r}$: Rear-wheel slip angle
$$\beta $$: Sideslip angle
${F}_{Zi}$: Vertical load on the ${i}^{th}$ wheel
$l$: Longitudinal wheel-base
$h$: Height of gravity center
${K}_{\varnothing f}$: Roll stiffness of the front suspension
${K}_{\varnothing r}$: Roll stiffness of the rear suspension
${C}_{\varnothing f}$: Roll damping ratio of the front suspension
${C}_{\varnothing r}$: Roll damping ratio of the rear suspension
${h}_{f}$: Roll center heights of the front axle
${h}_{r}$: Roll center heights of the rear axle
$LTR$: Load transfer ratio
$TTR$: Time to rollover
$T$: Single time step
${W}_{\beta }$: The weight coefficient of sideslip angle
${W}_{r}$: The weight coefficient of yaw rate
${W}_{LTR}$: The weight coefficient of $LTR$
${W}_{Fx}$: The weight coefficient of braking force
${r}_{d}$: Reference yaw rate
${\beta }_{d}$: Reference sideslip angle
$K$: Stability factor of the vehicle
$${LTR}_{d}$$: Reference $$LTR$$
${K}_{f}$: Cornering stiffness of the front axle
${K}_{r}$: Cornering stiffness of the rear axle
$\uppsi$: Braking force switching factor
$$k$$: Time step
${z}_{r}$: Driver's desired braking intensity
$${T}_{p}$$: Prediction step
$$z$$: Braking intensity
$s$: Sliding mode surface
$\Delta$: Thickness of boundary layer
${T}_{m}$: Motor maximum braking torque
