# Supplementary material for: Integrated control of braking-yaw-roll stability under steering-braking conditions
Source: Sci Rep. 2023 Nov 30;13:21110. doi: 10.1038/s41598-023-48535-1 (PMC10689841; doi:10.1038/s41598-023-48535-1)
Supplement: Supplementary file 1 — Supplementary Information. [file 41598_2023_48535_MOESM1_ESM.docx]

Integrated control of braking-yaw-roll stability under steering-braking conditions

Jia Chen^1,*^, Yihang Liu^2^, Renping Liu^2^, Feng Xiao^2^ and Jian Huang^2^

^1^ Automotive Engineering School, Chengdu Aeronautic Polytechnic, Chengdu 610100, China

^2^ College of Mechanical and Vehicle Engineering, Chongqing University, Chongqing 400044, China.

* Corresponding author: chenjia421@163.com

**Appendix A. Asymptotic stability of NMPC.**

Consider the following discrete-time nonlinear system^1-4^:

$x\left( k+1 \right)=F\left( x\left( k \right),u\left( k \right) \right)$ (A1)

where $x\left( k \right)\in\mathcal{X}$ represents the system state, $u\left( k \right)\in\mathcal{U}$ represents the vector of manipulation input, and $x_{0}$ is the initial condition. At the same time, the following assumptions are satisfied:

(1) $F\left( \cdot\right)$ is continuously differentiable in $\mathcal{X}\subset\mathcal{R}^{\boldsymbol{n}_{\boldsymbol{x}}}$ and $\mathcal{U}\subset\mathcal{R}^{\boldsymbol{n}_{\boldsymbol{u}}}$.

(2) The set $\mathcal{U}$ is compact and contains the origin.

(3) The set $\mathcal{X}\subset\mathcal{R}^{\boldsymbol{n}_{\boldsymbol{x}}}$ is control positive invariant for $F\left( \cdot\right)$, i.e. for $x\left( k \right)\in\mathcal{X}$ and $u\left( k \right)\in\mathcal{U}$, there is $F\left( x\left( k \right),u\left( k \right) \right)\in\mathcal{X}$. In addition, the set $\mathcal{X}$ is compact and contains the origin.

(4) Origin is an equilibrium state of the system.

(5) State $x\left( k \right)$ is measurable.

At time $k$, let $U_{\left[ k,k+N-1 \right]}\equiv\left\{ u\left( k \right),u\left( k+1 \right),\cdots,u\left( k+N-1 \right) \right\}$ represents a set of future inputs. Then, for system (A1), the cost function of nonlinear model predictive control is designed as follows:

$J\left( x\left( k \right),U_{\left[ k,k+N-1 \right]} \right)=\sum_{i=k}^{k+N-1} l\left( z\left( i \right),u\left( i \right) \right)+V_{f}\left( z\left( k+N \right) \right)$ (A2)

where $l\left( z\left( i \right),u\left( i \right) \right)=\left( {z\left( i \right)}^{T}W_{x}z\left( i \right)+{u\left( i \right)}^{T}W_{u}u\left( i \right) \right)$ and $V_{f}\left( z\left( i \right) \right)={z\left( i \right)}^{T}Pz\left( i \right)$; $W_{x}$ and $W_{u}$ are the control weights, which is called the positive definite weight matrix

$\begin{matrix} z\left( i+1 \right)=F\left( z\left( i \right),u\left( i \right) \right),\mathrm{for}k\leq i\leq k+N-1 \\ z\left( k \right)=x\left( k \right),z\left( i \right)\in\mathcal{X,}u\left( i \right)\in\mathcal{U,}z\left( k+N \right)\in\Omega\end{matrix}$ (A3)

where $N$ is the length of prediction and control time domain;$l\left( \cdot\right)$ is the cost term; $V_{f}\left( \cdot\right)$ is the terminal cost function; $P$ is the terminal weighting matrix of symmetric positive definite; $\left\{ z\left( i \right):k<i\leq N \right\}$ is the state predicted by the internal model; and the set $\Omega$ represents the terminal area near the origin. A fictitious local linear state feedback controller is selected to control the nonlinear system. The gain matrix of the controller is $L$. The terminal cost item satisfies $\forall z\left( k+N \right)\in\Omega$:

$V_{f}\left( z\left( k+N \right) \right)\geq\sum_{i=k+N}^{\infty} l\left( z\left( i \right),u\left( i \right) \right)$ (A4)

$u\left( i \right)=-Lz\left( i \right)\in\mathcal{U}$，$\left( k+N \right)\leq i\leq\infty$ (A5)

For the given optimal input $U_{\left[ k,k+N-1 \right]}^{*}\equiv\left\{ u^{*}\left( k \right),u^{*}\left( k+1 \right),\cdots,u^{*}\left( k+N-1 \right) \right\}$, the optimal prediction state sequence is:

$$z^{*}\left( k+j+1 \right)=F\left( z^{*}\left( k+j \right),u^{*}\left( k+j \right) \right)$$

Exist,

$J^{*}\left( x\left( k \right),{U^{*}}_{\left[ k,k+N-1 \right]} \right)=\sum_{i=k}^{k+N-1} \left( \left| z^{*}\left( i \right) \right|_{W_{x}}^{2}+\left| u^{*}\left( i \right) \right|_{W_{u}}^{2} \right)+\left| z^{*}\left( k+N \right) \right|_{P}^{2}$ (A6)

It can be obtained from literature^5^ that:

${\left| z\left( k+N+1 \right) \right|_{P}^{2}\leq\left| z^{*}\left( k+N \right) \right|}_{P}^{2}-\left| z^{*}\left( k+N \right) \right|_{Q^{*}}^{2}$ (A7)

$$J\left( x\left( k+1 \right),U_{\left[ k+1,k+N \right]} \right)=J^{*}\left( x\left( k \right),{U^{*}}_{\left[ k,k+N-1 \right]} \right)-\left( \left| x\left( k \right) \right|_{W_{x}}^{2}+\left| u^{*}\left( k \right) \right|_{W_{u}}^{2} \right)+\left| z\left( k+N \right) \right|_{Q^{*}}^{2}$$

$+\left| z\left( k+N+1 \right) \right|_{P}^{2}-\left| z^{*}\left( k+N \right) \right|_{P}^{2}$ (A8)

Combining Eq (A7) and (A8), we get

$J\left( x\left( k+1 \right),U_{\left[ k+1,k+N \right]} \right)\leq J^{*}\left( x\left( k \right),{U^{*}}_{\left[ k,k+N-1 \right]} \right)-\left( \left| x\left( k \right) \right|_{W_{x}}^{2}+\left| u^{*}\left( k \right) \right|_{W_{u}}^{2} \right)$ (A9)

Let ${U^{*}}_{\left[ k+1,k+N \right]}$ represent the discrete optimal solution, the optimality of the solution means

$J^{*}\left( x\left( k+1 \right),{U^{*}}_{\left[ k+1,k+N \right]} \right)\leq J\left( x\left( k+1 \right),U_{\left[ k+1,k+N \right]} \right)$ (A10)

Combining Eq (A9) and (A10), we get

$J^{*}\left( x\left( k+1 \right),{U^{*}}_{\left[ k+1,k+N \right]} \right)-J^{*}\left( x\left( k \right),{U^{*}}_{\left[ k,k+N-1 \right]} \right)\leq-\left( \left| x\left( k \right) \right|_{W_{x}}^{2}+\left| u^{*}\left( k \right) \right|_{W_{u}}^{2} \right)$ (A11)

Due to $W_{x}>0$ and $W_{u}>0$, then $\left( \left| x\left( k \right) \right|_{W_{x}}^{2}+\left| u^{*}\left( k \right) \right|_{W_{u}}^{2} \right)>0$, so $J^{*}\left( x\left( k \right),{U^{*}}_{\left[ k,k+N-1 \right]} \right)$ is a strictly decreasing function.

Define a Lyapunov function:

$$V\left( x\left( k \right) \right)\equiv J^{*}\left( x\left( k \right),{U^{*}}_{\left[ k,k+N-1 \right]} \right)$$

From Eq. (A6), we can get

$\lambda_{min}\left( W_{x} \right)\left| x\left( k \right) \right|^{2}\leq V\left( x\left( k \right) \right)=\sum_{i=k}^{k+N-1} \left( \left| z^{*}\left( i \right) \right|_{W_{x}}^{2}+\left| u^{*}\left( i \right) \right|_{W_{u}}^{2} \right)+V_{f}\left( x\left( k \right) \right)$ (A12)

then

$\eta\left( x\left( k \right) \right)\leq V\left( x\left( k \right) \right)$, $\forall x\left( k \right)\in\Omega$, with $\eta\left( x\left( k \right) \right)=\lambda_{min}\left( W_{x} \right)\left| x\left( k \right) \right|^{2}$

In addition, by Eq (A11)-(A12), we get

$V\left( x\left( k+1 \right) \right)-V\left( x\left( k \right) \right)<-\eta\left( x\left( k \right) \right)$ (A13)

Eq.(A13) shows that the nominal system is actually exponentially stable at the origin. Then the discrete-time NMPC is asymptotically stable at the origin, that is, when $k\to\infty$, $\left| x\left( k \right) \right|\to0$.

**Appendix B. Stability analysis of sliding mode controller.**

Lemma: for Lyapunov function $V$: $\left[ 0,\infty\right]\in R$, the solution of the inequality equation $\dot{V}=-\alpha V+f$, $\forall t\geq t_{0}\geq0$ is

$V\left( t \right)\leq e^{-\alpha\left( t-t_{0} \right)}V\left( t_{0} \right)+\int_{t_{0}}^{t} e^{-\alpha\left( t-\tau\right)}f\left( \tau\right)d\tau$ (B1)

where $\alpha$ is an arbitrary constant. The proof process of the above lemma is as follows:

Let $\omega\left( t \right)\triangleq\dot{V}+\alpha V-f$, then $\omega\left( t \right)\leq0$, and

$\dot{V}=-\alpha V+f+\omega\left( t \right)$ (B2)

Then, the solution is

$V\left( t \right)=e^{-\alpha\left( t-t_{0} \right)}V\left( t_{0} \right)+\int_{t_{0}}^{t} e^{-\alpha\left( t-\tau\right)}f\left( \tau\right)d\tau+\int_{t_{0}}^{t} e^{-\alpha\left( t-\tau\right)}\omega\left( \tau\right)d\tau$ (B3)

Since $\omega\left( t \right)<0$, $\forall t\geq t_{0}\geq0$, then

$V\left( t \right)\leq e^{-\alpha\left( t-t_{0} \right)}V\left( t_{0} \right)+\int_{t_{0}}^{t} e^{-\alpha\left( t-\tau\right)}f\left( \tau\right)d\tau$ (B4)

If $f=0$, the solution of $\dot{V}\leq-\alpha V$ is

$V\left( t \right)V\left( t \right)\leq e^{-\alpha\left( t-t_{0} \right)}V\left( t_{0} \right)$ (B5)

If $\alpha$ is a positive real number, it converges exponentially to zero. The exponential reaching law of the slip surface $s$ is designed as

$\dot{s}=-\varepsilon\mathrm{sgn}\left( s \right)-k_{d}s$, $\varepsilon>0, k_{d}>0$

where $\dot{s}=-k_{d}s$ is the exponential approach term and its solution is $s=s\left( t_{0} \right)e^{-k_{d}t}$. The Lyapunov function $V=\frac{1}{2}s^{2}$ is defined, and the exponential reaching law is adopted to obtain

$\dot{V}\leq-\varepsilon\left| s \right|-k_{d}s^{2}=-\frac{k_{d}}{2}V-\varepsilon\left| s \right|\leq-\frac{k_{d}}{2}V$ (B6)

Using lemma, the above formula $\dot{V}\leq-\frac{k_{d}}{2}V$, with $\alpha=\frac{k_{d}}{2}$, $f=0$ can be solved as

$V\left( t \right)\leq e^{-\frac{k_{d}}{2}\left( t-t_{0} \right)}V\left( t_{0} \right)$ (B7)

It can be seen from the above equation that $V\left( t \right)$ converges to zero in the form of exponential, that is, $s\to0$, and the convergence speed depends on $k_{d}$.

**References**

1 A, C. R., B, D. W. G., C, S. C. P., B, L. T. B. & A, H. K. P. Terminal region characterization and stability analysis of discrete time quasi-infinite horizon nonlinear model predictive control. *Journal of Process Control* **83**, 30-52 (2019).

2 Chen, H., Allg & #Xf, F. A Quasi-Infinite Horizon Nonlinear Model Predictive Control Scheme with Guaranteed Stability. *Automatica (Journal of IFAC)* (1998).

3 Biegler, L. T., Yang, X. & Fischer, G. A. G. Advances in sensitivity-based nonlinear model predictive control and dynamic real-time optimization. *Journal of Process Control* **30**, 104-116 (2015).

4 Qin, S. J. & Badgwell, T. A. Constrained model predictive control: Stability and optimality. *Control Engineering Practice* **11**, 733-764 (2003).

5 Rawlings, J., Mayne, D. & Diehl, M. Model Predictive Control: Theory, Computation, and Design. (2017).
